# Anemia risk in relation to lead exposure in lead-related manufacturing

**DOI:** 10.1186/s12889-017-4315-7

**Published:** 2017-05-05

**Authors:** Nan-Hung Hsieh, Shun-Hui Chung, Szu-Chieh Chen, Wei-Yu Chen, Yi-Hsien Cheng, Yi-Jun Lin, Su-Han You, Chung-Min Liao

**Affiliations:** 1 Department of Veterinary Integrative Biosciences, College of Veterinary Medicine and Biomedical Sciences, Texas A&M University, College Station, TX 77845 USA; 2 Institute of Labor, Occupational Safety and Health, Ministry of Labor, New Taipei City, 22143 Taiwan, ROC; 3 Department of Public Health, Chung Shan Medical University, Taichung, 40242 Taiwan, ROC; 4 Department of Family and Community Medicine, Chung Shan Medical University Hospital, Taichung, 40242 Taiwan, ROC; 5 Department of Biomedical Science and Environmental Biology, Kaohsiung Medical University, Kaohsiung, 80708 Taiwan, ROC; 6 Institute of Computational Comparative Medicine, Department of Anatomy and Physiology, College of Veterinary Medicine, Kansas State University, Manhattan, Kansas, 66506 USA; 7 Department of Bioenvironmental Systems Engineering, National Taiwan University, Taipei, 10617 Taiwan, ROC; 8 National Environmental Health Research Center, National Health Research Institutes, Miaoli County, 35053 Taiwan, ROC

**Keywords:** Blood lead, Benchmark dose, Probabilistic risk assessment, Health management, Decision analysis

## Abstract

**Background:**

Lead-exposed workers may suffer adverse health effects under the currently regulated blood lead (BPb) levels. However, a probabilistic assessment about lead exposure-associated anemia risk is lacking. The goal of this study was to examine the association between lead exposure and anemia risk among factory workers in Taiwan.

**Methods:**

We first collated BPb and indicators of hematopoietic function data via health examination records that included 533 male and 218 female lead-exposed workers between 2012 and 2014. We used benchmark dose (BMD) modeling to estimate the critical effect doses for detection of abnormal indicators. A risk-based probabilistic model was used to characterize the potential hazard of lead poisoning for job-specific workers by hazard index (HI). We applied Bayesian decision analysis to determine whether BMD could be implicated as a suitable BPb standard.

**Results:**

Our results indicated that HI for total lead-exposed workers was 0.78 (95% confidence interval: 0.50–1.26) with risk occurrence probability of 11.1%. The abnormal risk of anemia indicators for male and female workers could be reduced, respectively, by 67–77% and 86–95% by adopting the suggested BPb standards of 25 and 15 μg/dL.

**Conclusions:**

We conclude that cumulative exposure to lead in the workplace was significantly associated with anemia risk. This study suggests that current BPb standard needs to be better understood for the application of lead-exposed population protection in different scenarios to provide a novel standard for health management. Low-level lead exposure risk is an occupational and public health problem that should be paid more attention.

**Electronic supplementary material:**

The online version of this article (doi:10.1186/s12889-017-4315-7) contains supplementary material, which is available to authorized users.

## Background

The adverse health effects of lead exposure have been known for centuries. Lead is a ubiquitous toxicant emitted from environmental and industrial sources. Exposure routes of lead uptake in the workplace include ingestion and inhalation of inorganic lead. Therefore, the major sources of lead exposure may come from dust, fume, and poor personal hygiene including eating and smoking through contaminated hands. Many biomonitoring methods are capable of measuring the bioaccumulation of lead in the human body [1]. Lead levels in bone, blood, urine, and exfoliated teeth are the major biomarkers of internal dose. Nowadays, blood lead (BPb) measurement is still the primary technique to investigate recent lead accumulation in the body. Recently, lead condensate in exhaled breath was also shown to be a suitable non-invasive biomarker of occupational exposure in recent years [2].

Lead poisoning is considered the most well characterized occupational disease. The inhaled/ingested lead can transport to the heart, bones, intestines, kidneys, reproductive, and nervous systems, causing tissue-specific adverse effects [3, 4]. Implements of industrial hygiene and control measures have significantly decreased workers’ BPb concentrations over the last few decades [5, 6]. However, recent studies indicate that lead-exposed workers may suffer from adverse health effects under the current standards [7–9]. Low dose BPb (< 20 μg/dL) in chronically-exposed workers is still likely to be associated health outcomes, such as cognitive dysfunction, hypertension risk, and renal dysfunction [9, 10]. In recent years, these low-dose toxic effects of lead have received considerable attention, thereby questioning the capability of the current standard for critical dose of lead to protect the health of lead-exposed population. The issue not only in lead industries, but it also causes some problems in public health in several areas [11–14].

Adverse hematological effects are some of the manifestations of lead poisoning in lead workers. Chronic lead poisoning inhibits the ability to produce hemoglobin by interfering with enzymatic steps in the heme synthesis pathway and diminishes red blood cells, thereby increasing risk of anemia [15]. The absorption of lead can cause iron deficiency and may further cause anemia. Anemia associated with chronic lead exposure is a result of both interferings with heme biosynthesis and by decreasing red blood cell survival.

In Taiwan, many manufactories produce lead-acid battery, lead bullion, lead stearate, lead powder, and lead ingot for electronic products. Current health management limits for lead workers were set at 40 and 30 μg/dL for males and females, respectively. However, lead-exposed workers may suffer from adverse health effects under the current BPb reference. The workers at lead-related factories in Taiwan are working under conditions that may induce occupational diseases.

The main purpose of this study was to assess the risk of anemia for workers in lead factories and to provide a suggested BPb limit to improve health management for lead workers. A probabilistic risk assessment approach was used to estimate the BPb effect doses for abnormal hematological indicators and to assess anemia risk associated with exposure to lead. Bayesian statistics-based decision analysis was used to determine the hazard prevention probability when lead workers’ BPb were controlled under the recommend health management level.

## Methods

### Study population

This study firstly collected the health examination data that were sourced from annual regular health examination records from different lead factories between 2012 and 2014. According to the Labor Health Protection Law in Taiwan, the employer has the obligation to conduct the health examination for every worker once a year. Lead workers also need to receive the special physical and medical examinations. The examination items were included the inspection of BPb and hematological indicators of hematocrit (Hct), hemoglobin (Hgb), red blood cell count (RBC), mean corpuscular volume (MCV), mean corpuscular hemoglobin (MCH), and mean corpuscular hemoglobin concentration (MCHC) [16]. Some other information such as gender, birthdate, start working date and manufacturing area were also been recorded. Based on the Labor Health Protection Law, lead workers were asked to complete the questionnaire of special physical and the medical examination that included five items of demographic data, working experience, past medical history, living habits, and self-perceived symptoms. To characterize exposure history, we collected birth dates, dates of employment and examination records from the subjects. Work experience was used to describe the past and current working processes to further characterize exposure history. According to these information, the job-specific exposure conditions were calculated based on the working areas for each lead worker.

The total of 533 male and 218 female lead-exposed workers’ data were collected to construct the dataset, sourcing from 6 factories with different manufactory types (Table 1). The average ages of male and female workers were 43.7 (sd: 10.2) and 47.1 (8.6) yrs. All 533 male workers were working in the lead battery, lead stearate, and lead bullion factories, respectively. All 218 female workers were working in lead battery factories. Within a lead battery factory, the manufacturing areas were classified as casting, grinding, powder, filling, formatting, cutting, assembling, charging, packaging, or other off-site positions. The job-specific exposure conditions were calculated based on the working areas for lead workers.

**Table 1:** Basic statistics of age, working years, BPb levels, and indicators of hematopoietic function for 533 male and 218 female lead-exposed workers

Item	Unit	Mean (SD)	Min – Max
Basic information			
Age	yrs	43.7 (10.2) / 47.1 (8.6)^a^	21–65 / 27–64
Working years	yrs	11.8 (10.9) / 13.4 (9.7)	0–38 / 1–42
Blood lead	μg/dL	21.4 (13.3) / 11.3 (5.7)	2.1–101.4 / 1.8–31.3
Hematopoietic function			
Hematocrit	%	44.8 (3.2) / 40.1 (3.6)	31.3–57.8 / 26.6–46.9
Hemoglobin	g/L	15.11 (1.2) / 13.1 (1.4)	10.5–18.8 / 7.9–15.5
Red blood cell count	×10^6^ /μL	5.16 (0.50) / 4.6 (0.4)	3.73–7.44 / 3.6–6.4
Mean corpuscular volume	fl	87.4 (7.1) / 87.6 (8.6)	57–102 / 49.4–97.1
Mean corpuscular hemoglobin	pg	29.5 (2.9) / 28.7 (3.4)	17.5–38.6 / 14.1–33.9
Mean corpuscular hemoglobin concentration	%	33.8 (1.3) / 32.7 (1.3)	29.2–38.9 / 28.1–36.1

^a^Male/Female

### Benchmark dose-based effect analysis

We used the health examination record to construct the dose-response relationships between BPb levels and adverse hematological indicators. We used correlation analysis is the preliminary exam in effect analysis that allows us to understand the strength between the effect factors and anemia-associated indicators. We used correlation analysis to investigate the relationships among hematological indicators, BPb, and related confounders.

Hematological indicators have the reference ranges in the clinical medicine that can be used to diagnose the conditions of hematopoietic system. This study adopted these reference ranges to calculate the number and propotion of normal and abnormal lead workers in male and female populations. For estimating the critical effect dose of BPb for lead-associated abnormal hematological indicator, we employed the benchmark dose (BMD) models that have been used in many previous risk assessment studies [16].

We calculated the dichotomous response from quantal data using BMD models, as summarized in Table S1 (see Additional file 1). The benchmark response (BMR) was set to an abnormal proportion of 10% (BMR_10_) in the dose-response relationshi, as a conservative approach to protect the health of lead-exposed.

### Rick characterization

Health risk of anemia for workers at job-specific working areas was calculated by using a joint probability function combining the predicted BMD_10_ as acceptable risk limits and BPb concentration. We used a Monte Carlo (MC) approach to generate the large number of simulated samples of BPb and to understand the most likely BPb value in each job category. We considered the potential sources of variability and uncertainty in the BPb distributions as a probability density function based on measured values.

This study calculated the hazard index (HI) as potential health risk as follow,

1 $$ \mathrm{P}\left(\mathrm{HI}\right)=\frac{\mathrm{P}\left(\mathrm{BPb}\right)}{\mathrm{P}\left(\mathrm{BMD}\right)}. $$

The estimated BMDs were further treated probabilistically by considering the BMD_10_ and BMDL_10_, representing the mean and lower levels of 95% confidence interval (CI), respectively. HI > 1 implies that unacceptable health risk of anemia will occur for lead-exposed workers in the specific working group and HI < 1 indicates acceptable health risk with the proportion of health effect <10%.

Total risk (TR) can be estimated by the HI for each job category as,

2 $$ \mathrm{TR}=\sum_{\mathrm{i}=1}^{\mathrm{n}}\left({HI}_{\mathrm{i}}\times {p}_{\mathrm{i}}\right), $$

where *p*_i_ is the proportion of lead-exposed worker in the specific job category. Thus, hazard occurrence probability can be calculated by using the proportion of simulated HI which value is greater than 1.

The risk contribution of job (R_job_, %) can be estimated as,

3 $$ {\mathrm{R}}_{\mathrm{job}}=\frac{{\mathrm{HI}}_{\mathrm{i}}\times {p}_{\mathrm{i}}}{\mathrm{TR}}. $$

### Bayesian decision analysis

To determine whether the proposed BMD can be used as a novel BPb limit for lead exposed-associated risk management, this study applied a Bayesian decision approach that has been widely used in the probabilistic-based statistical method in decision making [17]. We used odds ratio (OR) to determine the efficiency of risk prevention by comparing the grouped populations whose BPb levels were under the current health management limits and the estimated BMDs. After that, this study conducted the product of binomial likelihood that sourced from collected health examination data as *Y*_0_ ~ binomial (*π*_0_,  *n*_0_) and *Y*_1_ ~ binomial (*π*_1_,  *n*_1_) with the parameters of interest as *θ* = (*π*_0_,  *π*_1_) where *Y*_0_ and *Y*_1_ are the observed normal and abnormal individuals in indicators of hematopoietic function. The parameters of *π*_0_ and *π*_1_ are the ratio of normal and abnormal hematological indicators in the subpopulation number of *n*_0_ and *n*_1_, respectively. The OR was estimated as,

4 $$ \mathrm{OR}=\frac{\pi_1\left(1-{\pi}_0\right)}{\pi_0\left(1-{\pi}_1\right)}. $$

We assumed the beta priors for the occurred probabilities *π*_0_ and *π*_1_ with parameters (*a*_0_, *b*_0_) and (*a*_1_, *b*_1_), respectively. The posterior can be given by (*π*_0_| *y*) ~ beta (*y*_0_ + *a*_0_,  *n*_0_ + *b*_0_) and (*π*_1_| *y*) ~ beta (*y*_1_ + *a*_1_,  *n*_1_ + *b*_1_). Estimation of the posterior distribution of ORs can be simply obtained by simulating the distribution of *f*(*π*_0_| *y*) and *f*(*π*_1_| *y*). Finally, hazard prevention probability was calculated by using the proportion of simulated ORs. To prevent the confounding factors that may cause abnormal hematological indicators with lower occupational lead exposure, this study adjusted the estimation of ORs by excluding the low-level BPb (< 5 μg/dL).

### Uncertainty and data analysis

Statistics and regressions of this study were conducted in the open source language R (Version 3.1.1, The R Foundation for Statistical Computing). The *p*-value <0.05 was considered to be statistically-significant. The BMD models were performed by BMD software (Version 2.5.1, USEPA) and followed the guidelines from USEPA [18, 19]. The Cochran-Armitage test for trend analysis was used to assess the presence of a dose-response relationship. We chose the dose-response dataset with *p*-value <0.05 to perform subsequent data analysis.

The best-fitted model and associated BMD and BMDL for each dose-response relation were selected based on the Akaike’s information criterion (AIC) and *p*-value of Chi-squared (χ^2^). The AIC was defined as penalized likelihood function as *AIC* =  − 2 log *L* + 2*p* that *p* was the number of parameters in the model and *L* is the maximum likelihood value.

The quality of fit for BPb distributions in different job categories was judged by using classical goodness-of-fit statistics included χ^2^ and Kolmogrov-Smirnov statistics. This study took into account all BMD models that had large *p*-value from Pearson χ^2^ goodness-of-fit statistics. To integrate the risk estimates across different models, the model-averaging BMD was applied by calculating the weight *w*_k._ for each model as [20],

5 $$ {w}_k=\frac{ \exp \left(-0.5{AIC}_k\right)}{\sum_{i=1}^K \exp \left(-0.5{AIC}_i\right)}. $$

Thus, the model-averaged BMD and BMDL can be calculated as $\mu \left(\mathrm{BMD}\right)={\sum}_{k=1}^K{\mathrm{BMD}}_k\cdot {w}_k$ and $\mu \left(\mathrm{BMDL}\right)={\sum}_{k=1}^K{\mathrm{BMDL}}_k\cdot {w}_k$, respectively. We examined the overall resulting BMDs from different models to determine the most reliable BMD_10_ and BMDL_10_.

Uncertainty is a key factor in risk assessment that can influence the precision of risk estimation [21]. This study considered the uncertainty and its impact on the expected risk estimates that were quantified by MC simulation. Each simulation was carried out with 10,000 iterations to assure the stability of its probability distribution.

## Results

### BPb distribution

**Fig. 1:**
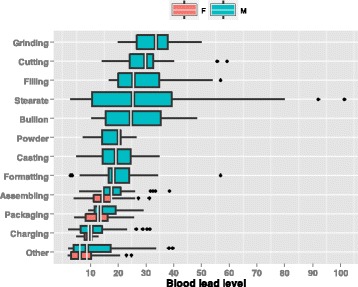
Boxplots ordering the job-specific BPb levels for male and female workers in lead factories

Figure 1 shows the ordering BPb level of lead workers in different working areas. Male workers in the lead battery manufacturing processes of grinding, cutting, and filling had relatively higher BPb concentrations than those in other working areas. Median BPb concentrations were estimated 24.5 and 25.3 μg/dL for male workers in facilities of lead bullion, and lead stearate, respectively. The highest BPb concentration was observed in lead stearate factories. Compared with male workers, female workers in lead battery factories had lower BPb levels.

The distributions of the abnormal number in hematological indicators are shown in Table 2. We found out that the abnormal proportion of indicator can exceed 20% for male workers in the exposure groups of grinding, powder, formatting, assembling, and charging, whereas the abnormal proportion may exceed 20% for female workers in every working group. Overall, the abnormal proportions ranged between 0 and 37.5% for each subgroup.

**Table 2:**
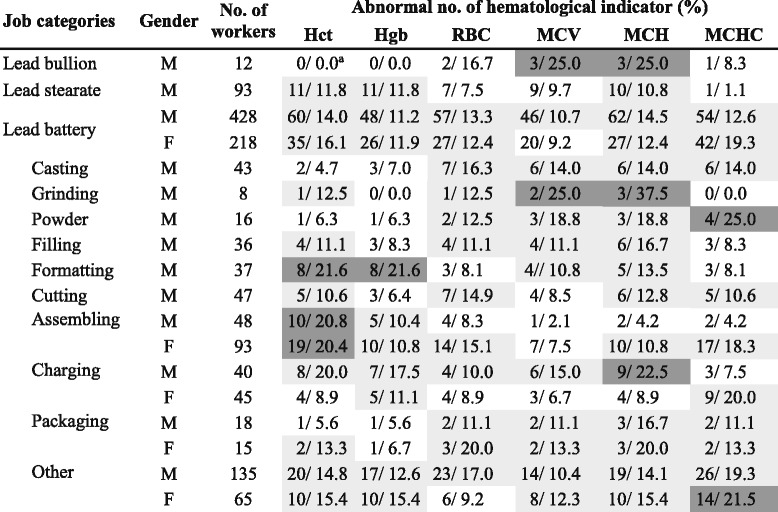
Job-specific number of workers and the related abnormal number of hematological indicators in each job

^a^Percentage (%)

(white: <10%, light gray: 10–20%, and dark gray: > 20%)

### Critical effect dose estimation

**Fig. 2:**
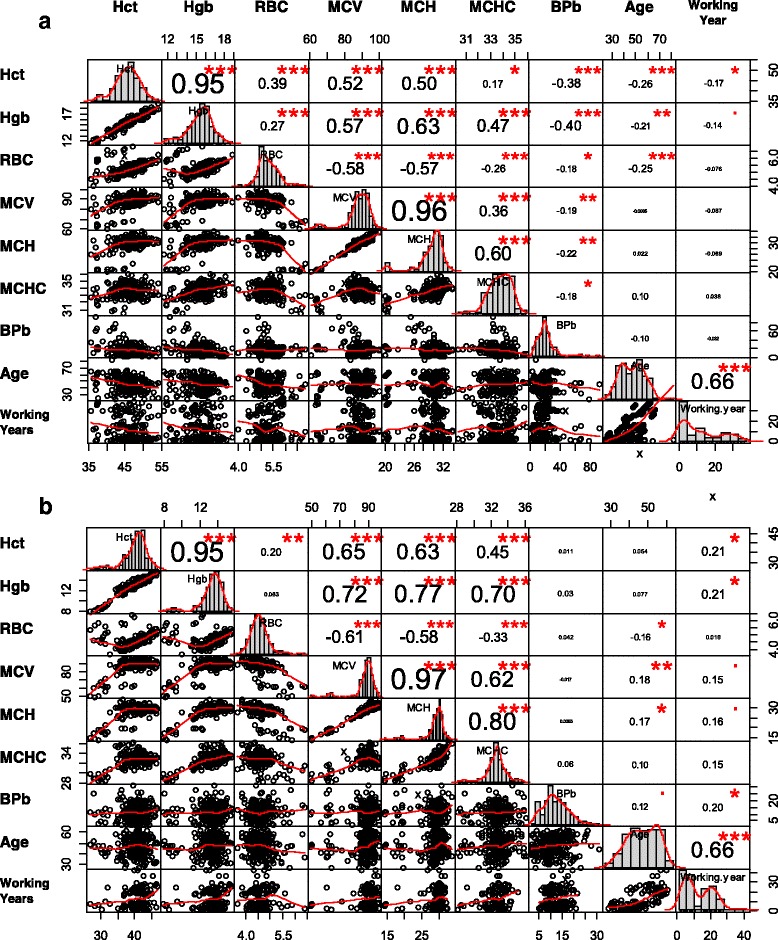
Correlation matrix showing the relations among BPb level, age, working years, and indicators of hematopoietic function for lead-exposed workers for (**a**) male and (**b**) female (represented as Pearson correlation coefficient: * *p* < 0.05, ** *p* < 0.01, *** *p* < 0.001)

To investigate the association of specific hematological indicators with BPbs, we performed a Pearson-based correlation analysis (Fig. 2). The BPb was significantly correlated with all hematological indicators, including Hct (*r* = −0.38; *p* < 0.001), Hgb (*r* = −0.40; *p* < 0.001), RBC (*r* = −0.18; *p* < 0.05), MCV (*r* = −0.19; *p* < 0.01), MCH (*r* = −0.22; *p* < 0.01), and MCHC (*r* = −0.18; *p* < 0.05) for male workers (Fig. 2a).

To determine the critical effect dose, we used BMD modeling to estimate the BPb levels that could associate with 10% abnormal proportion of each hematological indicator. Through Cochran-Armitage trend test, our results found that hematological indicators had significant dose-response trend with BPb concentration in male workers, including Hct (χ^2^ = 3.336; *p* < 0.01), Hgb (χ^2^ = 1.741; *p* < 0.05), MCV (χ^2^ = 3.879; *p* < 0.001), and MCH (χ^2^ = 3.343; *p* < 0.001) (see Additional file 2: Tables S2 – S5). Among female workers, hematological indicators had significant dose-response trend for Hct (χ^2^ = 2.110; *p* < 0.05), RBC (χ^2^ = 1.849; *p* < 0.05), MCV (χ^2^ = 1.887; *p* < 0.05), and MCH (χ^2^ = 2.472; *p* < 0.01) (see Additional file 2: Tables S6 – S9).

The model averaging BMD_10_ estimates were 53.5 (BMDL_10_: 40.5), 60 (44.5), 37.8 (22.1), and 42.0 μg/dL (28.8) for Hct, Hgb, MCV, and MCH for male workers, respectively (Tables S2 – S5). Among female workers, model averaging BMD_10_ estimates were 8.6 (BMDL_10_: 5.5), 9.8 (6.3), 12.1 (9.4), and 10.7 μg/dL (7.9) for Hct, RBC, MCV, and MCH, respectively (Tables S6 – S9).

**Fig. 3:**
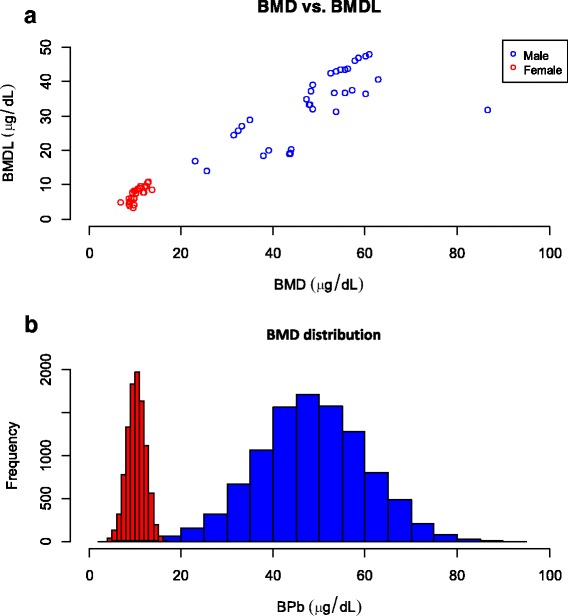
**a** Scatter plot showing the relationships between effect doses of BMD and BMDL_10_ for male and female. **b** Distribution of BMD of BPb for male and female lead workers

Figure 3a shows that BMD_10_ were strongly correlated with BMDL_10_ for male (*r* = 0.69) and female (*r* = 0.82) workers. Based on MC simulation, the BMDs were depicted as a probability density function of normal distribution that average BMD_10_ were 48.39 ± 11.58 (mean ± sd) and 10.26 ± 1.98 μg/dL for male and female workers, respectively (Fig. 3b).

### Anemia risk estimates

**Fig. 4:**
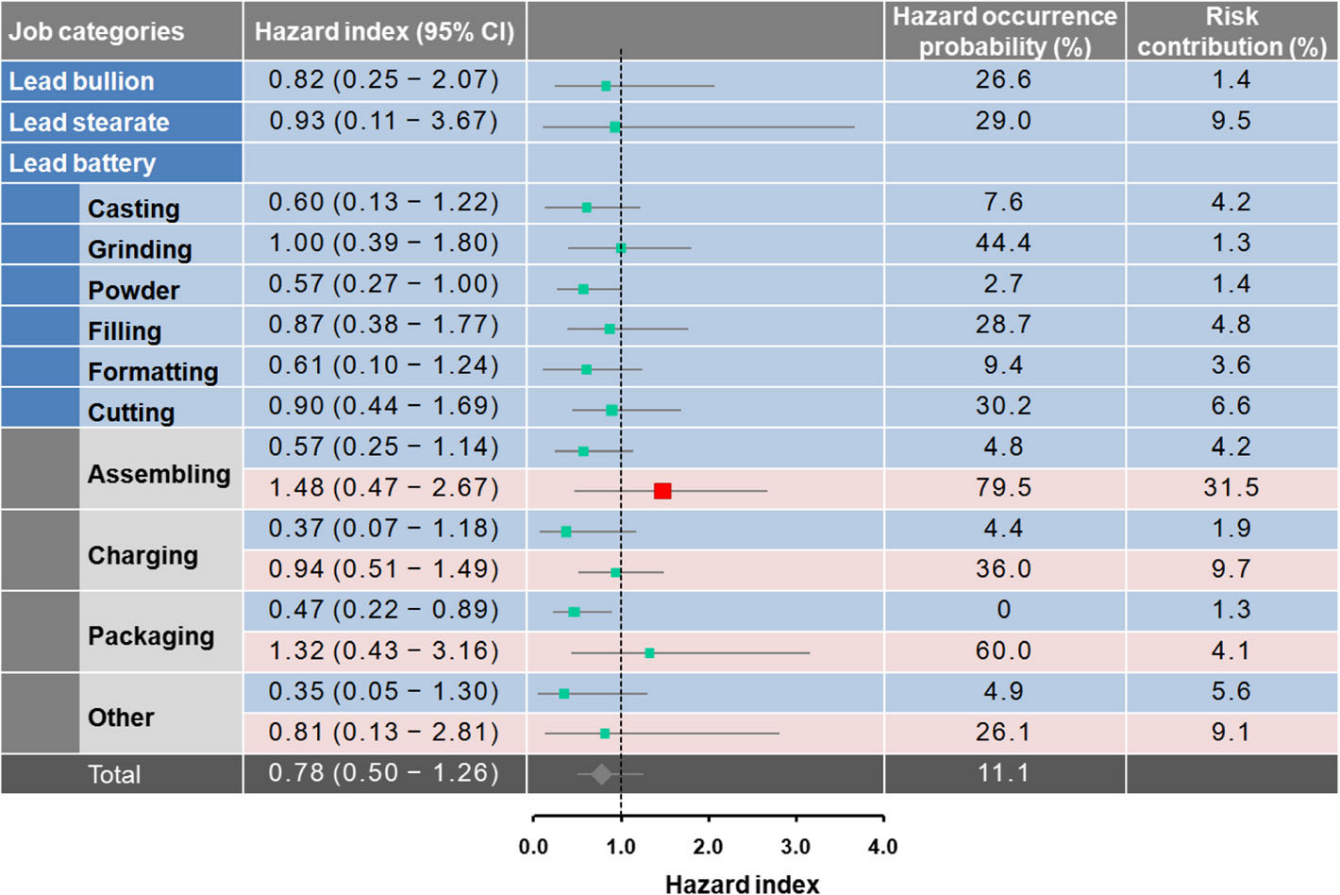
Forest plot showing the estimated hazard index with 95% confidence intervals and relative risk contribution for each working group. Female workers are represented by light gray

We found that the highest hazard occurrence probability was in male grinding workers with median HI of 1.00 (95% CI: 0.39–1.80) (Fig. 4). For female workers, assembling and packaging work groups had relatively higher hazard occurrence probabilities of 80% and 60% with the median HIs of 1.48 (0.47–2.67) and 1.32 (0.43–3.16), respectively. The overall risk in HI for lead-exposed workers was 0.78 (0.50–1.26) with hazard occurrence probability of 11%. Risk contribution analysis indicated that female workers in the assembling group contributed a significant high risk to the total population, with a contribution proportion of 31.5%. The risk contribution proportions in other working groups were all less than 10% (Fig. 4).

### Health management-based risk decision

Based on the probability distribution (Fig. 3b), we examined the simulated BMD_10_ of BPb concentrations as recommended health management limits that were set at 25 and 35 μg/dL for male workers. The recommended standards for female workers were set at 10 and 15 μg/dL. We used the recommended BMDs to compare with the current health management limits that were 40 and 30 μg/dL for male and female workers, respectively.

**Fig. 5:**
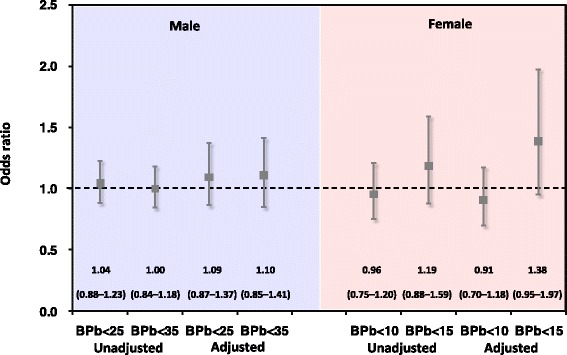
Bayesian analysis-based ORs determining the available of calculated BMD_10_ as novel BPb limit. Bars indicate 95% confidence intervals. Dotted line, null value (OR = 1.0)

Figure 5 shows the results of decision analysis. Confounding was addressed using data stratification. ORs and adjusted ORs in male workers were 1.00 (95% CI: 0.84–1.18) and 1.10 (0.85–1.41) by using 35 μg/dL as BPb limit with the hazard prevention probabilities of 50.6% and 76.2% under unadjusted and adjusted scenarios, respectively. Moreover, when we adopted 25 μg/dL as BPb limits, the results of ORs and adjusted ORs were 1.04 (0.88–1.23) and 1.09 (0.87–1.37) with the hazard prevention probabilities of 67.0% and 76.9% under unadjusted and adjusted scenarios, respectively. ORs and adjusted ORs were 1.19 (0.88–1.59) and 1.38 (0.95–1.97) for female workers by using 15 μg/dL as BPb limit with the hazard prevention probabilities of 85.5% and 95.0% under unadjusted and adjusted scenarios, respectively (Fig. 5). It can be seen that estimated ORs were relatively higher when we adopted 15 μg/dL as BPb limit. The estimated ORs and adjusted ORs, respectively, were 0.96 (0.75–1.20) and 0.91 (0.70–1.18) when using 10 μg/dL as BPb limit. The hazard prevention probabilities were estimated 32.0% and 22.9% under unadjusted and adjusted scenarios, respectively.

## Discussion

### Risk quantification and decision

This study focuses on lead exposure-related anemia risk. Therefore, we used hematological indicators to represent BPb-induced anemia. The Hct, Hgb, and RBC were representative indicators of lead-associated occupational anemia and can be used to evaluate the critical BPb levels to protect lead workers [16]. In addition to common indicators, our study also applied the measurements of MCV, MCH, and MCHC, which were calculated by three original indicators to estimate the possible critical values.

This study found that BPb-related hematological effects of anemia were consistent with previous studies [16, 22]. Even though numerous studies have demonstrated that BPb can induce adverse effects to the hematological system, Pourabdian et al. [23] found that indicators of Hgb, MCV, and white blood cell cell did not display concentration-dependence on BPb levels. Some confounders such as menstruation, smoking, and working status may influence the relationships between BPb and hematological indicators.

In this study, the BMD model gave us an opportunity to estimate the critical effect dose for BPb-associated anemia in workers. BMD modeling has been utilized as a more precise mathematical approach for performing toxicological risk assessment [24]. Compare to the traditional method of using NOAEL (No observed adverse effect level) or LOAEL (Lowest observed adverse effect level), BMD modeling can be further applied to model prediction in the toxicological and epidemiological investigation.

Overall, the BMD models provide more accurate and precise value by using the computational approach, which considers the uncertainty from the epidemiological information and mathematical models. Our results show that BMD_10_ and BMDL_10_ estimates ranged from 23 to 87 and 15–48 μg/dL for male workers after adjusting for of age and working years. Our estimation of BMD_10_ was similar to those reported by Karita et al. [16], which ranged from 48 to 73 μg/dL. Further, our BMDL_10_ estimates were consistent with their results, which ranged from 33 to 49 μg/dL.

According to the recent statement from American College of Occupational and Environmental Medicine (ACOEM), BPb is still a reliable and wide-used biomonitoring indicator for the health effects associated with lead exposure. The most compelling epidemiology evidence shows that the increased cardiovascular morbidity and mortality in populations with BPb in the low to medium range of 10 to 20 μg/dL [25, 26]. The most notable effect is the increased cardiovascular risks at these BPb levels, but the adverse pregnancy outcomes and other effects may also occur.

Risk assessment results demonstrated that lead workers are likely to work in unsafe workplaces. Unacceptable hazard occurrence probability was approximately 11.1% for total lead workers. Female workers in the assembling group had a higher risk of 79.5%, resulting in hematological effects, which may exceed the acceptable level. Risk contribution was 31.5% within total lead workers. However, the highest hazard contribution in male lead workers was estimated at only 9.5% in the grinding group. It seems that appropriate engineering control measures and hygiene strategies still play an important role in limiting lead exposure for female lead workers [27].

To determine whether the estimated critical values of BPb can be used as the novel limit for health management, this study successfully applied a Bayesian analysis approach integrated with MC simulation to estimate the uncertainty and reliability of risk control efficiency. Our approach had also been widely used in exposure analysis and risk decision-making in environmental and occupational health research [28, 29].

To determine the BPb levels in health management, we examined the simulated BPbs that were set at 25 and 35 μg/dL for male workers. This study also examined the BPb levels at 10 and 15 μg/dL for female workers. Our results found out that there were slight differences when considering these two BPb levels for male workers. Although the ORs and hazard prevention probabilities of a BPb level at 25 μg/dL were higher than those of a BPb level at 35 μg/dL, it seems that the improving efficiency had reached the theoretical level. Therefore, the suitable BPb level of male workers for health management of anemia can be set in this interval. The BPb-hematological BMDs for female workers were lower than male, indicating that female workers are more vulnerable for lead-related anemia than male. We found that BPb level at 15 μg/dL could be used as a suitable health management limited rather than 10 μg/dL.

Our results also revealed that 95% CIs of Bayesian analysis-based ORs all cover the value of 1, indicating that the risk control efficiency may be slightly insignificant. However, the results from hazard prevention probabilities showed that there were nearly 50–70% probabilities of improving the lead-related anemia by restraining BPb limit up to 25–35 μg/dL for male workers. The hazard prevention probabilities for female workers were 85.5–95.0% when BPb limit of 15 μg/dL was adopted. Thus, the anemia risk can also be reduced when a more stringent BPb level is adopted.

### Limitations and implications

Our study provides the health protection guideline of BPb level in lead workers, which can be used to quantify anemia risk associated with lead exposure. This study did not focus on the exposure assessment of airborne lead. Compared with environmental monitoring of airborne lead concentration and permissible exposure level, BPb is more reliable and can be used to determine gender-specific health effects of lead. In addition, the airborne lead cannot fully reflect the health risk if the company successfully implements personal protection equipment and industrial hygiene.

This study could not investigate and control the other possible sources of lead exposure outside the work and get the complete information on previous experience of lead-related work. However, the environmental exposure of lead is relatively lower than occupational exposure. This study focuses on the dose-response relationship between BPb concentration and the hematological indicators from regular health examination. The BPb was mainly sourced from the current workplace. Therefore, we assumed that the cumulative BPb that sourced from workplace or other area can be associated with the change of the hematological indicators.

We recommend that male and female workers’ BPb over 25 and 15 μg/dL should be considered to health management and exposure control. Lead-exposed workers need to be continuously controlled and reduce their BPb even if levels are under the current limits of 40/30 μg/dL for male/female workers. Flora et al. [30] reported that BPb levels of 10–20 μg/dL could precipitate anemia. The possible threshold of lead-associated anemia was also proposed by previous studies, which was approximately 20 μg/dL for lead workers [11, 16]. By contrast, our study found that BPb-related hematological effects may display gender-specificity due to inter-individual variability. Furthemore, there is no evidence of a safe exposure level for renal and cardiac effects associated with exposure to lead [31, 32].

According to our correlation analysis, we found that BPb had significantly negative correlations with hematological indicators in male workers. However, this correlation was not observed in female workers. This result might due to the fact that female workers had relatively lower BPb levels compared to males. Moreover, we were unable to collect information about the menstruation or pregnancy from our collated data for female workers. This data gap may influence our risk estimation results. The constructed dose-response relationship was an atypical S-shape in our effect analysis. We observed some abnormal hematological indicators in female workers who had lower BPb concentrations, an effect which may have been due to menstruation or pregnancy. Nonetheless, BPb levels in female workers needs to be carefully revised in order to prevent the lead-related anemia risk.

In addition to hematological effects of anemia, health effects of low-dose lead also include hypertension, cognitive dysfunction, renal, and reproductive effects. Lead may also be genotoxic [33, 34]. Neuropsychological effects of lead toxicity were also determined to be a common occupational hazard of lead toxicity [35]. Neurotoxic effects in lead-exposed workers were observed at BPb level below 20 μg/dL, which was a more sensitive toxicity phenotype compared to other lead-induced adverse effects. Ahmad et al. [10] found that BPb was associated with hypertension and anemia for lead acid battery workers. Therefore, lead workers might suffer from illnesses associated with low-level lead exposure.

Computational approaches are a useful research tool in the health risk assessment of chemical exposure, including heavy metal [36]. A computational modeling approach was used to estimate airborne lead concentrations by using BPb levels [37]. OEHHA [37] used a computational approach and recommended that the BPb level should be between 5 to 10 μg/dL for workers over 40 years of working in a factory. Thus, the 8-h time-weighted-average airborne lead concentrations must not exceed 2.1 μg/m^3^. The permissible exposure limit (PEL) is 50 μg/m^3^ in Taiwan. Therefore, stringent PEL and personal hygiene strategy should be considered to reduce exposure to airborne lead.

Our study found that female workers had lower lead effect dose than male workers, suggesting that, compared to males, female workers are likely to have a higher risk of lead-induce anemia. In Taiwan, the Occupational Safety and Health Act suggests that pregnant women should stop working in the current lead-exposed workplace and prohibits them to work until one year after childbirth. Kosnett [8] recommended that women who are or may become pregnant and have a BPb > 5 μg/dL should reduce their exposure to lead.

This study applied the model averaging method to integrate BMD estimates across multiple models. It has been used as an effective method for estimating the model uncertainty in BMD estimation. The model averaging method has also been widely applied to dichotomous dose-response relationships in a variety of risk assessment contexts such as occupational and epidemiological studies [38, 39]. However, several limitations may affect the accuracy of BMD estimation. Wheeler and Bailer [38] indicated that the “average-model” method often failed to adequately cover the true BMD when a linear or near linear dose-responses was modeled. The model averaging and the corresponding BMD and BMDL are potentially biased in low-dose scenarios.

The observed average BPb levels were much lower than the current health management limits in this study. However, this study still found some workers with hematological effects even though the BPb were under the permissible level. The proportions of lead working population with abnormal hematological indicators were estimated >10% in each job category. Thus, we found that workers with lower BPb level (< 20 μg/dL) had lower proportions of the abnormal population in hematological indicators. We also found out that some lead workers may still work in an unsafe condition with risk estimates of HI > 1, especially for female workers. It would be better to establish a new occupational strategy and enhance the education and training to reduce the BPb concentrations and subsequent hematotoxicity. Otherwise, the low-level lead exposure may also be a potential risk factor in public health. Our research framework can also be applied to assess other lead induced-adverse health effects in exposed population. Furthermore, a long-term follow-up investigation is urgently needed to characterize lead-associated adverse health effects.

## Conclusions

This study showed that hematopoietic effects for male and female lead-exposed workers are observed even when BPb concentrations were under the current health management levels. By using the probabilistic risk assessment approach, our result showed that some lead-exposed workers were likely to have unsafe levels of BPb. The BMDs for lead exposed-induced health effects provided the novel limits on BPb for health management. Our results suggest that the current BPb limit needs to be better characterized to better protect the health of lead-exposed population. Low-level lead exposure risk is an occupational and public health problem that should be paid more attention.

## Additional files

Additional file 1: Table S1. Summary of the Benchmark dose (BMD) models used in this study. (DOCX 32 kb)

Additional file 2: Table S2-S9. Fitted quantal model for male lead-exposed workers. (DOCX 27 kb)

## Abbreviations

BMD: Benchmark dose
BMR: Benchmark response
BPb: Blood Lead
Hct: Hematocrit
Hgb: Hemoglobin
HI: Hazard index
LOAEL: Lowest observed adverse effect level
MC: Monte Carlo
MCH: Mean corpuscular hemoglobin
MCHC: Mean corpuscular hemoglobin concentration
MCV: Mean corpuscular volume
NOAEL: No observed adverse effect level
OR: Odds ratio
PEL: Permissible exposure limit
RBC: Red blood cell
TR: Total risk
