# Supplementary material for: Anemia risk in relation to lead exposure in lead-related manufacturing
Source: BMC Public Health. 2017 May 5;17:389. doi: 10.1186/s12889-017-4315-7 (PMC5420139; doi:10.1186/s12889-017-4315-7)
Supplement: Supplementary file 1 — Summary of the Benchmark dose (BMD) models used in this study. (DOCX 32 kb) [file 12889_2017_4315_MOESM1_ESM.docx]

**Table S1**

Summary of the Benchmark dose (BMD) models used in this study

| Model | Risk function, *R*(*d*) |  | Constraints |
| --- | --- | --- | --- |
| Dichotomous Hill | **$vg+\frac{v\left(1-g\right)}{1+\exp\left(-\beta_{0}-\beta_{1}\log d\right)}$** | (T1) | 0 ≤ *g* < 1, 0 < *v* ≤ 1, *β* ≥ 0 |
| Gamma | **$\gamma+\frac{1-\gamma}{\Gamma\left(\alpha\right)}\int_{0}^{\beta_{1}d} t^{\alpha-1}e^{t}dt$** | (T2) | *α* ≥ 0, *β* > 0, 0 ≤ *γ* < 1 |
| Logistic | **$\frac{1}{1+\exp(-\beta_{0}-\beta_{1}d)}$** | (T3) | *β* > 0 |
| Loglogistic | **$\gamma+\frac{1-\gamma}{1+\exp\left(-\beta_{0}-\beta_{1}\log d\right)}$** | (T4) | 0 ≤ *γ* < 1, *β* > 0 |
| Logprobit | **$\gamma+\left(1-\gamma\right)\Phi\left(\beta_{0}+\beta_{1}\log d\right)$** | (T5) | 0 ≤ *γ* < 1, *β* > 0 |
| Multistage | **$\gamma+\left(1-\gamma\right)\left[1-\exp\left(-\sum_{i=1}^{j} \beta_{j}d^{j}\right)\right]$** | (T6) | 0 ≤ *γ* < 1, *β*_j_ ≥ 0 |
| Probit | **$\Phi(\beta_{0}+\beta_{1}d)$** | (T7) | *β* > 0 |
| Quantal-linear | **$1-\exp(-\beta_{0}-\beta_{1}d)$** | (T8) | *β* ≥ 0 |
| Weibull | $\gamma+(1-\gamma)[1-\exp(-\beta_{1}d^{\alpha})]$ | (T9) | *α* ≥ 0, *β* > 0 |

Parameter meanings: *d* is the dose; *α* is the power; *β*_0_ is the intercept; *β*_1_ is the slope; γ is the background; *v* is the maximum probability of response predicted by the dichotomous Hill model; *g* multiplied by *v* is the background probability of response.
