# Supplementary material for: Anemia risk in relation to lead exposure in lead-related manufacturing
Source: BMC Public Health. 2017 May 5;17:389. doi: 10.1186/s12889-017-4315-7 (PMC5420139; doi:10.1186/s12889-017-4315-7)
Supplement: Supplementary file 2 — Fitted quantal model for male lead-exposed workers. (DOCX 27 kb) [file 12889_2017_4315_MOESM2_ESM.docx]

**Table S2**

Fitted quantal model for BPb-related abnormal Hct for male lead-exposed workers

| **Fitted model** | ***p*-value for χ^2^** | **AIC** | **Weights** | **BMD_10_** | **BMDL_10_** |
| --- | --- | --- | --- | --- | --- |
| **χ^2^ =3.336, *p*=0.004** | | | | | |
| Gamma | 0.4445 | 177.34 | 0.134 | 54.57 | 43.4 |
| Logistic | 0.3579 | 177.61 | 0.117 | 48.57 | 38.92 |
| Probit | 0.3103 | 178.13 | 0.091 | 48.22 | 37.3 |
| Loglogistic | 0.4408 | 177.37 | 0.132 | 55.61 | 43.54 |
| Logprobit | 0.4456 | 177.34 | 0.134 | 53.75 | 42.85 |
| Multistage | 0.3361 | 177.96 | 0.099 | 53.29 | 36.63 |
| Weibull | 0.4419 | 177.37 | 0.132 | 56.26 | 43.85 |
| Quantal-Linear | 0.1197 | 180.54 | 0.027 | 53.6 | 31.29 |
| Dichotomous Hill | 0.4408 | 177.37 | 0.132 | 55.61 | 36.78 |
| **“Average-model” model averaging** | | | | **53.53** | **40.45** |

**Table S3**

Fitted quantal model for BPb-related abnormal Hgb for male lead-exposed workers

| **Fitted model** | ***p*-value for χ^2^** | **AIC** | **Weights** | **BMD_10_** | **BMDL_10_** |
| --- | --- | --- | --- | --- | --- |
| **χ^2^ =1.741, *p*=0.0408** | | | | | |
| Gamma | 0.5129 | 246.48 | 0.178 | 58.62 | 46.91 |
| Logistic | 0.1971 | 248.74 | 0.057 | 57.25 | 37.39 |
| Probit | 0.1801 | 248.97 | 0.051 | 60.16 | 36.54 |
| Loglogistic | 0.5018 | 246.57 | 0.170 | 60.01 | 47.29 |
| Logprobit | 0.5177 | 246.43 | 0.182 | 57.8 | 46.19 |
| Multistage | 0.2652 | 247.86 | 0.089 | 62.89 | 40.57 |
| Weibull | 0.4985 | 246.6 | 0.168 | 60.83 | 47.88 |
| Quantal-Linear | 0.1181 | 249.78 | 0.034 | 86.58 | 31.75 |
| Dichotomous Hill | 0.3845 | 248.35 | 0.070 | 52.43 | 42.47 |
| **“Average-model” model averaging** | | | | 59.98 | 44.53 |

**Table S4**

Fitted quantal model for BPb-related abnormal MCV for male lead-exposed workers

| **Fitted model** | ***p*-value for χ^2^** | **AIC** | **Weights** | **BMD_10_** | **BMDL_10_** |
| --- | --- | --- | --- | --- | --- |
| **χ^2^ =3.879, *p*<0.0001** | | | | | |
| Gamma | 0.0745 | 279.93 | 0.078 | 43.77 | 19 |
| Logistic | 0.1478 | 278.2 | 0.186 | 34.96 | 28.79 |
| Probit | 0.1455 | 278.4 | 0.168 | 33.29 | 27.04 |
| Loglogistic | 0.0756 | 279.9 | 0.080 | 43.74 | 19.24 |
| Logprobit | 0.0690 | 279.99 | 0.076 | 43.96 | 20.21 |
| Multistage | 0.1448 | 278.31 | 0.176 | 37.91 | 18.42 |
| Weibull | 0.0784 | 279.89 | 0.080 | 43.62 | 19.06 |
| Quantal-Linear | 0.0963 | 279.99 | 0.076 | 22.96 | 16.86 |
| Dichotomous Hill | 0.0756 | 279.9 | 0.080 | 43.74 | 19.24 |
| **“Average-model” model averaging** | | | | 37.75 | 22.05 |

**Table S5**

Fitted quantal model for BPb-related abnormal MCH for male lead-exposed workers

| **Fitted model** | ***p*-value for χ^2^** | **AIC** | **Weights** | **BMD_10_** | **BMDL_10_** |
| --- | --- | --- | --- | --- | --- |
| **χ^2^ =3.343, *p*=0.0004** | | | | | |
| Gamma | 0.2314 | 333.67 | 0.135 | 47.75 | 33.42 |
| Logistic | 0.2282 | 333.85 | 0.123 | 32.5 | 25.83 |
| Probit | 0.2084 | 334.18 | 0.104 | 31.36 | 24.35 |
| Loglogistic | 0.2300 | 333.7 | 0.133 | 48.09 | 33.25 |
| Logprobit | 0.2288 | 333.66 | 0.135 | 47.3 | 34.81 |
| Multistage | 0.2504 | 333.49 | 0.147 | 39.01 | 20.12 |
| Weibull | 0.2341 | 333.71 | 0.132 | 48.56 | 32.13 |
| Quantal-Linear | 0.1120 | 335.97 | 0.043 | 25.65 | 14.05 |
| Dichotomous Hill | 0.1429 | 335.69 | 0.049 | 47.89 | 33.26 |
| **“Average-model” model averaging** | | | | 42.04 | 28.75 |

**Table S6**

Fitted quantal model for BPb-related abnormal Hct for female lead-exposed workers

| **Fitted model** | ***p*-value for χ^2^** | **AIC** | **Weights** | **BMD_10_** | **BMDL_10_** |
| --- | --- | --- | --- | --- | --- |
| **χ^2^ =2.110, *p*=0.0174** | | | | | |
| Gamma | 0.8969 | 117.05 | 0.113 | 8.75 | 5.23 |
| Logistic | 0.8611 | 117.23 | 0.104 | 9.87 | 8.3 |
| Probit | 0.8704 | 117.18 | 0.106 | 9.49 | 7.9 |
| Loglogistic | 0.8962 | 117.05 | 0.113 | 8.74 | 5.02 |
| Logprobit | 0.9052 | 117.01 | 0.116 | 8.74 | 3.86 |
| Multistage | 0.8904 | 117.07 | 0.112 | 8.8 | 5.22 |
| Weibull | 0.8929 | 117.07 | 0.112 | 8.74 | 5.22 |
| Quantal-Linear | 0.8366 | 116.12 | 0.180 | 6.84 | 4.87 |
| Dichotomous Hill | 0.7672 | 118.97 | 0.043 | 8.66 | 4.37 |
| **“Average-model” model averaging** | | | | 8.6 | 5.54 |

**Table S7**

Fitted quantal model for BPb-related abnormal RBC for female lead-exposed workers

| **Fitted model** | ***p*-value for χ^2^** | **AIC** | **Weights** | **BMD_10_** | **BMDL_10_** |
| --- | --- | --- | --- | --- | --- |
| **χ^2^ =1.849, *p*=0.0323** | | | | | |
| Gamma | 0.9407 | 103.53 | 0.109 | 9.76 | 6.22 |
| Logistic | 0.9303 | 103.59 | 0.105 | 11.03 | 9.2 |
| Probit | 0.9332 | 103.57 | 0.106 | 10.66 | 8.81 |
| Loglogistic | 0.9403 | 103.53 | 0.109 | 9.76 | 6.03 |
| Logprobit | 0.9420 | 103.53 | 0.109 | 9.69 | 3.25 |
| Multistage | 0.9394 | 103.54 | 0.108 | 9.81 | 6.22 |
| Weibull | 0.9402 | 103.53 | 0.109 | 9.79 | 6.22 |
| Quantal-Linear | 0.9049 | 102.25 | 0.206 | 8.6 | 5.91 |
| Dichotomous Hill | 0.8188 | 105.53 | 0.040 | 9.76 | 4.24 |
| **“Average-model” model averaging** | | | | 9.75 | 6.32 |

**Table S8**

Fitted quantal model for BPb-related abnormal MCV for female lead-exposed workers

| **Fitted model** | ***p*-value for χ^2^** | **AIC** | **Weights** | **BMD_10_** | **BMDL_10_** |
| --- | --- | --- | --- | --- | --- |
| **χ^2^ =1.887, *p*=0.0302** | | | | | |
| Gamma | 0.2724 | 76.83 | 0.085 | 12.28 | 9.72 |
| Logistic | 0.2354 | 77.19 | 0.071 | 12.9 | 10.91 |
| Probit | 0.2450 | 77.09 | 0.075 | 12.64 | 10.61 |
| Loglogistic | 0.2696 | 76.87 | 0.083 | 12.32 | 9.67 |
| Logprobit | 0.2830 | 76.74 | 0.089 | 12.2 | 9.7 |
| Multistage | 0.4178 | 75.04 | 0.208 | 12.48 | 9.6 |
| Weibull | 0.2664 | 76.9 | 0.082 | 12.36 | 9.68 |
| Quantal-Linear | 0.3078 | 76.23 | 0.115 | 13.63 | 8.62 |
| Dichotomous Hill | 0.4721 | 75.2 | 0.192 | 9.99 | 8.27 |
| **“Average-model” model averaging** | | | | 12.11 | 9.43 |

**Table S9**

Fitted quantal model for BPb-related abnormal MCH for female lead-exposed workers

| **Fitted model** | ***p*-value for χ^2^** | **AIC** | **Weights** | **BMD_10_** | **BMDL_10_** |
| --- | --- | --- | --- | --- | --- |
| **χ^2^ =2.472, *p*=0.0067** | | | | | |
| Gamma | 0.6059 | 98.85 | 0.053 | 11.81 | 7.78 |
| Logistic | 0.8042 | 96.82 | 0.145 | 11.15 | 9.52 |
| Probit | 0.8040 | 96.84 | 0.144 | 10.88 | 9.17 |
| Loglogistic | 0.6058 | 98.85 | 0.053 | 11.73 | 7.79 |
| Logprobit | 0.6044 | 98.86 | 0.052 | 11.9 | 7.82 |
| Multistage | 0.8527 | 95.22 | 0.324 | 10.21 | 7.41 |
| Weibull | 0.6083 | 98.83 | 0.053 | 11.7 | 7.79 |
| Quantal-Linear | 0.5044 | 97.15 | 0.123 | 9.16 | 6.23 |
| Dichotomous Hill | 0.6058 | 98.85 | 0.053 | 11.73 | 7.79 |
| **“Average-model” model averaging** | | | | 10.73 | 7.93 |
